# Soil horizons regulate bacterial community structure and functions in Dabie Mountain of the East China

**DOI:** 10.1038/s41598-023-42981-7

**Published:** 2023-09-22

**Authors:** Xia Luo, Yinping Gong, Feiyan Xu, Shuai Wang, Yingying Tao, Mengmeng Yang

**Affiliations:** School of Biological Science and Food Engineering, Chuzhou University, No. 1 Huifeng West Road, Chuzhou, 239000 Anhui China

**Keywords:** Forest ecology, Solid Earth sciences

## Abstract

Soil bacterial communities regulate nutrient cycling and plant growth in forests. Although these bacterial communities vary with soil nutrients and plant traits, the variation and degree with soil horizons in different forest types remain unclear. Here, bacterial communities of 44 soil samples from organic horizon (O horizon) and mineral horizon (M horizon) of three forest types (*Cunninghamia*, broad-leaved and *Pinus* forests) in subtropical forests of Dabie Mountain, China were analyzed based on amplicon sequencing. We assessed the effects of soil horizons and forest types on bacterial communities. The results showed that the bacterial richness and diversity were significantly higher in the O horizon than in the M horizon. Furthermore, the bacterial community composition and functions were also remarkably different between the two soil horizons. Furthermore, forest types could affect bacterial community composition but not for diversity and functions. Moreover, soil organic matter, including the total organic carbon, available phosphorus, total organic nitrogen, available potassium, ammonium nitrogen, and pH were main drivers for bacterial community composition. The results propose robust evidence that soil horizons strongly driven bacterial community composition and diversity, and suggest that microhabitat of soil bacterial communities is important to maintain the stability of forest ecosystem.

## Introduction

Soil microorganism play important roles in regulating critical terrestrial functions, nutrient cycling, and improving stability of ecosystem^1^. For example, soil bacterial communities are critical for soil nutrient transformations, nutrient and biogeochemical cycling, nitrogen cycling, carbon transformation and stabilization, and plant host defense^2–4^. Variations in soil bacterial communities represent the environment of soil resources and forest types. Moreover, some bacteria of soil pathogenic can affect the growth and development of plants, whereas many soil bacteria can improve the effective resource utilization and stress resistance of terrestrial plants^5^. In turn, soil bacterial communities mainly depend on soil nutrients, plant community composition and traits^6^. However, it remains largely unknown how forest types and soil properties in different horizons affect soil bacterial communities, thus presenting an important knowledge gap to understand plant–soil–microbe interactions.

Forests contribute 80% of the terrestrial biodiversity all over the world, and are complex network ecosystem of organisms including bacteria, fungi, plants, and animals. Forests especially for the changes of tree species compositions and diversity, can affect soil physical and chemical traits and nutrient cycling^3,7,8^, and then affect soil microbial communities by producing litter, rhizodeposition, and root symbiotic microorganisms^9^. Bacterial community function is closely associated with plant traits^10^. Thus, forest variation is ideal to study bacterial communities^11,12^. Previous research confirms that there is radical difference between broad leaf and coniferous forests based on soil bacterial community composition^6^. However, the effects of dominant tree species in a forest on soil bacterial communities are still a lot of unexplored. The different patterns of bacterial communities from soil horizons of different forest types should be predicted. Therefore, knowing whether the forest types cause changes in bacterial groups is necessary to bridge the knowledge gap in bacterial ecology.

Bacterial diversity and community composition are close relationships with soil characteristics^11^, such as pH, carbon to nitrogen ratio, carbon, nitrogen, NH_4_^+^-N, and nutrient availability^8,13,14^. Among these, pH of soil horizons can strongly predicts the diversity and structure of soil bacterial communities^15–17^. Some studies have shown that applying more nitrogen can significantly reduce soil bacterial diversity, whereas others show that nitrogen added have no significant effect on bacterial diversity^18,19^. The variations of forest types drive the changes of soil properties by tree establishment, growth, and mortality, but these changes also vary with soil horizons^8,20,21^. Microhabitat heterogeneity may affect the variability of bacterial decomposition^22^. However, it is undetermined whether the effect of soil properties rely on soil horizon to further drive bacterial communities. Investigating the effect of soil horizons on bacterial communities and their variations is important for better understanding of ecological interactions in a forest. The purpose of this study is to examine the effects of soil horizons and forest types on bacterial community structure in Dabie Mountain, China, using high-throughput Illumina sequencing. Specifically, the following hypothesis are explored: (1) bacterial diversity and richness would be decreased with soil horizons due to the content decreased of soil organic matter. (2) Bacterial community composition and function should be main driven by soil horizons. (3) Forest types could affect the bacterial community composition, but not for bacterial diversity and function.

## Results

**Figure 1:**
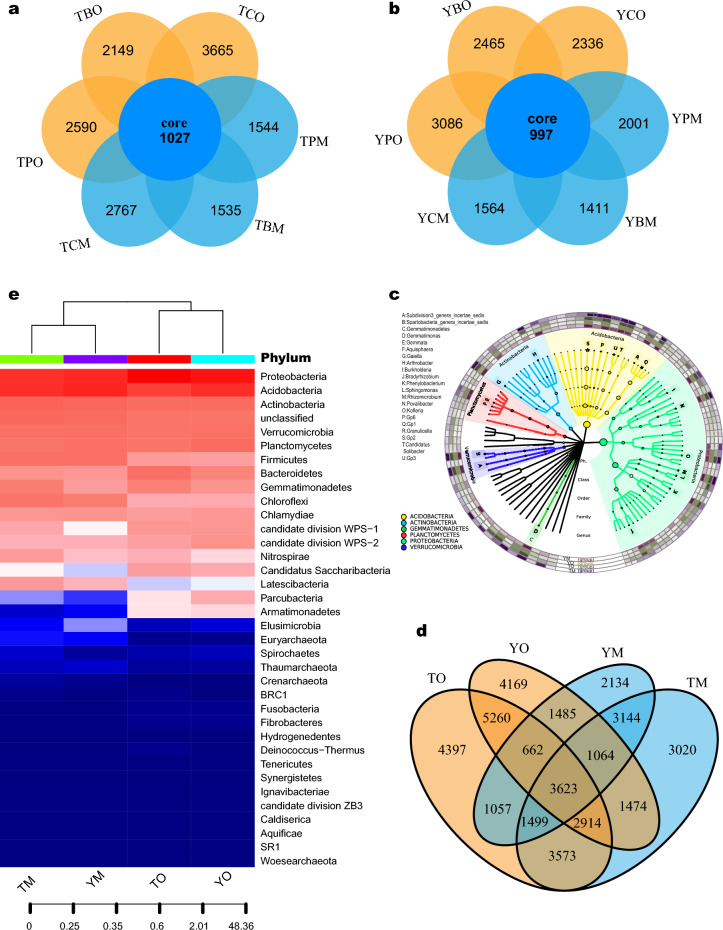
Venn diagrams of shared and unique bacterial OTUs in the O and M horizon and three forest types (**a**) at Tiantangzhai and (**b**) and Yaoluoping Nature Reserve (**d**) of Dabie Mountain. (**c**) Visualization of taxonomic and phylogenetic based on bacterial genus level by GraPhIAn. (**e**) The heatmap of bacterial community composition based on phylum level.

All soil samples were collected from two soil horizons in three forest types at two sites (i.e., TNR and YNR). Soil bacterial sequences were grouped into 8,405 OTUs. The most dominant bacterial phyla were Proteobacteria (37.36%) and Acidobacteria (30.04%), followed by Actinobacteria (7.72%) (Figs. 1). In addition, Alphaproteobacteria (21.01%) belonging to Proteobacteria was the most dominant class, whereas Cytophagia (0.24%) belonging to Bacteroidetes was the least dominant class. High number of sequences in the soil samples could not be classified at the genus level (36.66%).

### Effects of soil horizons on bacterial communities

**Figure 2:**
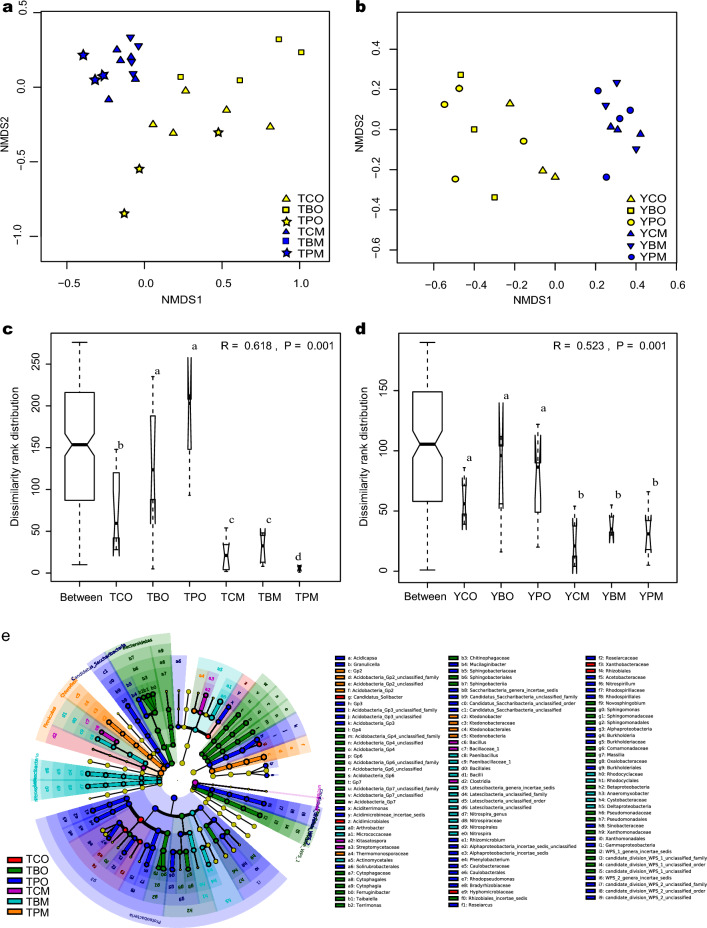

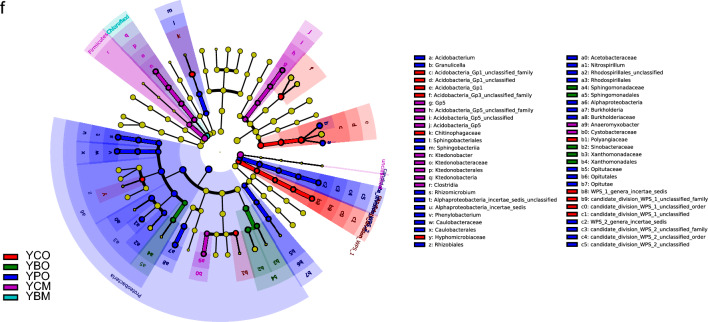
Nonmetric multidimensional scaling ordination (NMDS) of soil bacterial communities based on genus level across two horizons and three forest types (**a**) at Tiantangzhai and (**b**) Yaoluoping Nature Reserve based on the average Bray–Curtis dissimilarity matrix. The difference of bacterial community composition across different groups are analyzed (**c**) at Tiantangzhai and (**d**) Yaoluoping Nature Reserve. Yellow represents bacterial groups with no significant differences (**e**) at Tiantangzhai and (**f**) Yaoluoping Nature Reserve.

**Figure 3:**
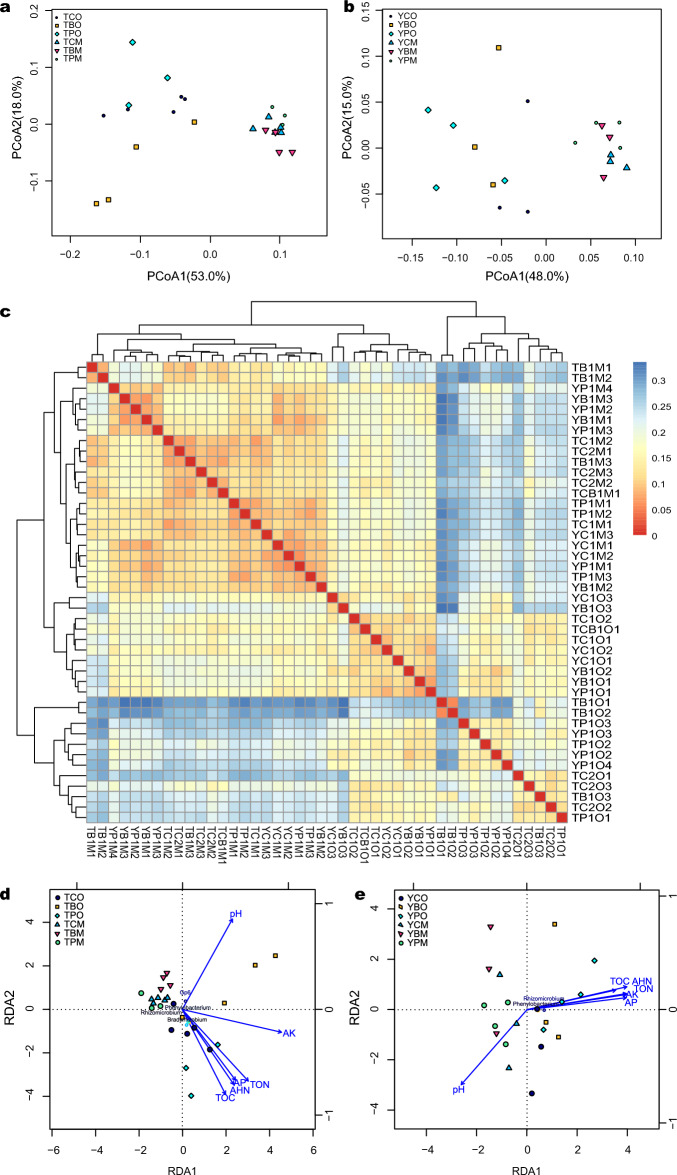
The effect of soil horizons and forest types on bacterial communities using principal coordinates analysis (PCoA) (**a**) at Tiantangzhai and (**b**) Yaoluoping Nature Reserve. (**c**) The similarity between samples is analyzed based on UniFrac distance. Ordination plots of the results from the redundancy analysis (RDA) used to explore the relationships between bacterial community and driving factors based on genus level (**d**) at Tiantangzhai and (**e**) Yaoluoping Nature Reserve.

Bacterial community composition varied according to soil horizons. Similar bacterial community composition was clustered because of the same soil horizon based on Hierarchical clustering analysis. 1027 and 997 OTUs were shared in all samples of subplots in three forest types of the two sites, respectively (Fig. 1a,b). The number of shared OTUs of the O and M horizon was 12,459 (31.56%) and 9330 (23.64%) in the two sites, respectively (Fig. 1d). Furthermore, the OTU abundance in the O horizon was higher than that in the M horizon. The soil bacterial richness and Shannon index were higher in the O horizon than in the M horizon in *Cunninghamia* and broad-leaved forests (Figs. 2). The dominant bacterial communities varied with soil horizons in the three forest types (Fig. 1c), and the two soil horizons shared 560 OTUs (Fig. 1d). For example, the abundance of Chloroflexi and Firmicutes was higher in the M horizon, whereas Proteobacteria and Bacteroidetes were higher in the O horizon (Fig. 1e). Notably, the bacterial community composition in the O horizon based on genus level was distinguished from the M horizon based on the NMDS analysis (Fig. 2a,b). The results of the PERMANOVA and ANOSIM analyses confirmed that bacterial community composition in the two horizons had significant differences in *Cunninghamia* and broad-leaved forests in TNR (PERMANOVA and ANOSIM, *P* < 0.05) and in *Pinus* forest in YNR (Fig. 2c,d). The different biomarkers of the O and M horizon were revealed based on LEfSe analysis (Fig. 2e,f, Figs. 3). Proteobacteria, Alphaproteobacteria, and Rhizobiales were main biomarkers in TO while Deltaproteobacteria, Micrococcaceae, and Arthrobacter were main biomarkers in TM. Planctomycetaceae, Rhodospirillales, and Planctomycetia were main biomarkers in YO while Acidobacteria were main biomarkers in YM. Soil bacterial community composition was markedly different between the two horizons in two sites. The bacterial composition in the O horizon was separated from that in the M horizon along the PCoA axis 1, which explained up to 48.0% and 53.0% of dissimilarity separately in YNR and TNR (Fig. 3a,b). PCoA showed that the O horizon differed from the M horizon, and the results were further proof of the remarkable effect of soil horizons on bacterial diversity.

### Effects of forest types on soil communities

Bacterial beta diversity based on OTUs was varied across the three forest types with no significant difference (Fig. 2a–d). Bacterial alpha diversity was also weakly affected by forest types although soil bacterial richness and Shannon indices were the lowest and highest in *Pinus* and *Cunninghamia* forests in TNR respectively (*P* < 0.05) (Figs. 2). However, the soil bacterial community structure in the O horizon in *Cunninghamia* forest was significantly different from *Pinus* and broad-leaved forests in TNR (PERMANOVA and ANOSIM, *P* < 0.05). LEfSe analysis revealed different biomarkers in the three forest types (Figs. 2e,f). Key bacterial groups varied with different forest types in the two sites, although groups such as *Kofleria*, *Candidatus Solibacter*, *Phenylobacterium*, *Burkholderia*, *Gaiella*, *Rhizomicrobium*, and *Gemmatimonas* were common genera in the three forest types. Furthermore, at the phylum level, the abundance of Parcubacteria and Fibrobacteres in the O horizon showed significant differences between *Cunninghamia* and broad-leaved forests in TNR. The abundance of Actinobacteria in the M horizon was the highest in *Cunninghamia* forest and the lowest in broad-leaved forest. The abundance of Bacteroidetes in the M horizon was the highest in *Cunninghamia* forest and the lowest in *Pinus* forest. At the genus level, *Acidipila*, *Acidobacterium*, and *Anaeromyxobacter* in the O horizon were the highest in *Cunninghamia* forest and the lowest in broad-leaved forest in YNR, and they showed no significant difference in the M horizon in YNR and in both horizons in TNR. These results suggested that forest types strongly effect soil bacterial community composition but they had no strong influence on bacterial beta- and alpha-diversity.

### Effects of soil properties on bacterial communities

The relationships between soil physical and chemical properties and bacterial composition of different samples with RDA analysis (Fig. 3d,e). The content of TOC, AP, TON, AK, and AHN in the O horizon was significantly higher than that in the M horizon. Furthermore, pH was the lowest in the *Pinus* forest than the *Cunninghamia* and broad-leaved forest although soil from the three forest types were acid soil (Table 1). The soil organic matter had no significant difference among the three forest types in O horizon, except that AP and TON were the lowest and highest in the *Pinus* forest in M horizon, respectively. There was positive relationship among TOC, AP, TON, AK, and AHN while they had negative relationship with pH. Meanwhile, TOC, AP, TON, AK, AHN, and pH were significant influences on bacterial community composition. For examples, TOC, AP, TON, AK, AHN, and pH were significantly related to the abundance of *Phenylobacterium* and *Rhizomicrobium* at the two sites. Moreover, TOC, AP, TON, AK, and AHN were significantly related to the abundance of Proteobacteria, Acidobacteria, and Actinobacteria, but pH did not significantly affect the three phyla. TOC, AP, TON, AK, AHN, and pH were significantly related to the abundance of Firmicutes and Armatimonadetes in YNR.

**Table 1:** Physical and chemical properties of soil at O and A horizon of forest types.

Parameters	TOC (%)	TON (mg/kg)	pH value	AHN (mg/kg)	AK (mg/kg)	AP (mg/kg)
TCO	12.88 ± 4.33	9131.40 ± 2071.5	4.95 ± 0.145	630.80 ± 161.06	277.60 ± 50.09	17.32 ± 7.14
TBO	7.26 ± 0.17	7151.25 ± 446.90	5.49 ± 0.26	498.25 ± 46.16	393.75 ± 47.46	9.18 ± 0.61
TPO	13.21 ± 7.21	9180.67 ± 2606.69	4.41 ± 0.33	640.67 ± 179.61	355.33 ± 96.46	12.96 ± 6.01
TCM	4.59 ± 0.68	4725 ± 224.44	5.02 ± 0.08	421.40 ± 61.82	149.40 ± 58.44	4.24 ± 1.03
TBM	4.11 ± 0.19	4689.50 ± 199.01	5.09 ± 0.10	347.25 ± 54.52	189.50 ± 37.42	2.95 ± 0.23
TPM	4.31 ± 0.35	3901.33 ± 259.33	4.86 ± 0.19	358.67 ± 22.28	184.33 ± 45.49	2.34 ± 0.25
YCO	9.18 ± 2.15	6165 ± 951.40	4.81 ± 0.24	504.33 ± 185.43	233.67 ± 7.57	8.82 ± 3.81
YBO	13.2 ± 2.47	8629.67 ± 1157.12	4.55 ± 0.18	710.33 ± 30.37	311 ± 38.43	11.97 ± 1.69
YPO	15.03 ± 7.43	9004.75 ± 2568.41	4.605 ± 0.25	707.25 ± 92.68	345 ± 112.13	16.925 ± 7.02
YCM	4.02 ± 0.96	3225.33 ± 725.90	4.98 ± 0.05	286.33 ± 77.70	121.67 ± 27.74	2.79 ± 0.88
YBM	3.51 ± 0.90	2656.33 ± 331.82	4.83 ± 0.11	242.00 ± 27.87	104.67 ± 14.01	1.89 ± 0.24
YPM	3.53 ± 0.75	2803 ± 524.19	4.8875 ± 0.16	222.26 ± 108.65	108 ± 8.49	2.45 ± 0.51

TCO, TBO, TPO, TCM, TBM, and TPM represent O and M horizon of three forest types respectively at Tiantangzhai nature reserve. YCO, YBO, YPO, YCM, YBM, and YPM represent O and M horizon of three forest types respectively at Yaoluoping nature reserve.

### Functional profiles of bacterial communities

**Figure 4:**
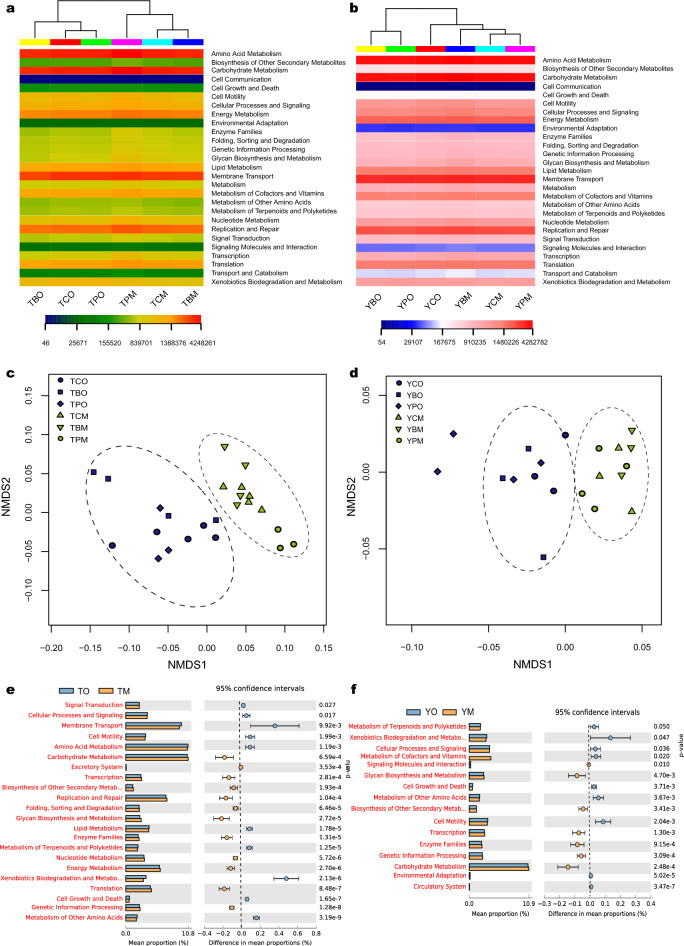
The relative functional abundance of soil bacterial communities based on heatmap of KEGG using hierarchical clattering and unweighted pair group method with arithmetic mean in the two horizons and three forest types (**a**) at Tiantangzhai and (**b**) Yaoluoping Nature Reserve. Nonmetric multidimensional scaling ordination (NMDS) of soil bacterial functional communities (**c**) at Tiantangzhai and (**d**) Yaoluoping Nature Reserve. Significantly altered bacterial communities between O and M horizon as measured by the response ratio method at the 95% confidence interval (Welch’s t-test) at (**e**) Tiantangzhai and (**f**) Yaoluoping Nature Reserve**.**

Metabolic functions of bacterial communities were predicted in KEGG pathways using PICRUSt software. The dominant metabolic functions of soil bacterial communities in the study sites were amino acid metabolism, circulatory system, replication and repair, and membrane transport. Hierarchical clustering analysis showed that bacterial community functions varied with soil horizons. The NMDS analysis also showed that bacterial community functions were driven by soil horizons (Fig. 4a,b). Metabolic functions, such as the metabolism of cofactors and vitamins, and membrane transport, were different between the O and M horizons, and they were significantly different in the O and M horizons of *Pinus* and broad-leaved forests in TNR. The bacterial community functions in the three forest types showed high similarity according to PCoA, especially in the M horizon (Fig. 4c,d). The abundance of biosynthesis of secondary metabolites, glycan biosynthesis and metabolism, carbohydrate metabolism, enzyme families, and transcription were higher in O horizon while the abundance of metabolism of terpenoids and polyketides, cell motility, and metabolism of other amino acids were higher in M horizon (Fig. 4e,f). Bacterial community functions in broad-leaved forest differed from *Pinus* forest in TNR, whereas they were clustered in YNR according to PCoA.

## Discussion

Forest ecosystems provide a broad range of habitats for bacteria, especially abundant in soil and litter. Bacteria play an important role in the transformation of dead plant biomass in litter and soil. Consistent with the first hypothesis, bacterial composition, diversity and functions varied with soil horizons, and the diversity in the O horizon were significantly higher than that in the M horizon. Our results confirmed that soil horizons strongly affect bacterial communities structure^20,23^. The distribution of bacterial communities is consistent with fungal communities in O and M horizon^24^. Soil that is a strongly spatial and temporal variability in biological, physical, and chemical properties, offers variety and complexity habitats for microbial organisms^20^. Soil horizons are an essential soil characteristic and the resultant of vegetation, climate, parent material, organisms, and time^25^. Soil properties in different horizons are main drivers for the changes of microbial community structure^26^. Further research showed that soil bacterial community composition was significantly influenced by soil organic matter content. Moreover, the content of TOC, AP, TON, AK, and AHN were significant higher in the O horizon than in the M horizon. This results highly suggest that TOC, AP, TON, AK, AHN, and pH in soil horizons contribute to construction of bacterial communities. We speculate that bacterial communities usually have high growth rates under nutrient rich microhabitat, such as nitrogen is critical for Proteobacteria^27^. Variations in bacterial communities with soil horizons are sensitive to the change of availability of soil organic matter^28^. The diversity and richness of bacterial communities are decreased with the content decreases of organic matter in the two horizons that strongly provide evidence.

Bacterial diversity is significantly associated with soil properties, especially carbon and nitrogen^6^. Bacterial carbon utilization can promote nitrogen availability, and then drive bacterial diversity and community construction^29^. Nitrogen tightly associates with soil organic matter and pH that indirectly affect bacterial communities^30^. This results suggest that the interaction of soil organic matter regulate the change of soil bacterial communities. Notably, soil pH is widely accepted as a main driver of variation in soil bacterial communities by influencing the bioavailability of carbon and nitrogen characteristic. Our study showed that soil pH of *Pinus* forest was the lowest and the bacterial richness and diversity were also the lowest among the three forests. The results indicate that the changes of soil microbial community composition are strongly correlated with pH^31^, and bacterial diversity is decreased with soil acidity^16^. Moreover, pH was negatively correlated with TOC, TON, AHN, AK, and AP, and strongly affected bacterial community composition according our results. For example, soil pH was significantly correlated with *Phenylobacterium* and *Rhizomicrobium*, and highly correlated with the diversity of dominant bacteria, including Acidobacteria, Alphaproteobacteria, Bacteroidetes, and Actinobacteria. These results further indicate that soil pH is a main predictor for bacterial diversity and community composition^17,29^. Studies of the indirect or direct effect by soil organic matter and pH on soil bacterial community composition can help understand the relationships between pH and microbial community.

Consistent with the third hypothesis, forest types affected bacterial community composition, and key bacterial groups varied with the three forest types although forest types weakly effect on bacterial diversity and bacterial community functions. In forest, tree species by litter quality, litter decomposition, shading, interception of precipitation, and windbreak directly affect the soil organic horizon and indirectly affect the mineral horizon, and then provide metabolic resources and microhabitat for microorganism^31,32^. Forest type dominated by specific tree species indirectly affect soil bacterial community composition and diversity by the effect on soil chemical properties and plants functional traits^9,30,33^. However, plant diversity and richness cannot drive the bacterial alpha diversity^34–36^. Our studies also dominate that forest types mainly effect on bacterial community composition rather than diversity.

The PICRUSt has recently become available for the determination of the metabolic and functional profiles in a broad range of host-associated microbial communities^37^. Soil bacterial communities play an important role in maintenance of ecosystem and sustainability. Our results suggest that the bacterial functional composition of O horizon is significantly different from M horizon. The content of soil organic matter is significant higher in the O horizon than in the M horizon (Table 1). These results indicate that higher soil organic matter could improve the soil bacterial functional community. Furthermore, the different of microhabitat with soil horizon significant effects on bacterial functions, and synergistic interactions among bacterial species and the composition of the bacterial community are important in determining the level of ecosystem functioning^38^. Notably, the functions, such as biosynthesis of secondary metabolites, glycan biosynthesis and metabolism, carbohydrate metabolism, enzyme families, and transcription are enriched in O horizon while the functions, such as metabolism of terpenoids and polyketides, cell motility and other amino acids are enriched in M horizon. Our studies showed that Proteobacteria and Acidobacteria were the dominant soil phyla and higher in O horizon than in M horizon. The two phyla contribute to nitrogen, carbon, and sulfur cycling^39–42^. Rhizobiales was main biomarker in organic matter according our results. Rhizobiales commonly exert beneficial functions for their hosts by providing various nutrients, phytohormons as well as precursors for essential plant metabolites, such as nitrogen fixing, methanotrophic, microsymbiotic bacteria^43^. Furthermore, the O horizon consist of undecomposed, partially, or highly decomposed litter composed and has high soil organic matter content^25^. The M horizon is characterized by an accumulation of humified organic matter mixed with the mineral fraction^25^. The bacterial activity contributes to decomposition and utilization of litter in O horizon while they might improve environmental adaptation of plant or their own^44^. The similarity functions are showed in the three forest types further indicate that soil organic matter in different horizon is main driver for bacterial communities rather than the type of litter. A limitation of this study is that only the effects of forest types on bacterial community composition were examined. Future research should consider plant species traits, richness and diversity to provide deeper insights into the mechanisms underlying the effects of forest types on the soil bacterial community structure and functions.

## Conclusions

This study reports the effects of soil horizons and forest types on bacterial communities in Dabie Mountain, China. The results suggest that dominant bacterial taxa playing different roles in the microenvironment varied with soil horizons and forest types. The diversity, composition and functions of bacterial communities were strongly correlated with soil horizon. Notably, forest types could affect soil bacterial community composition while had weak effect on bacterial diversity. Furthermore, our results highlight that soil organic matter and pH might concurrently determine the soil bacterial community structure. In conclusion, this study clarified that soil chemical characteristics in soil horizons were the main drivers of bacterial community structure. Further studies of relationships among plants, microbes, and soil are essential to reveal the mechanisms of the soil bacterial community structure.

## Materials and methods

### Site description

Two sites, Yaoluoping Nature Reserve (YNR) and Tiantangzhai Nature Reserve (TNR) respectively, were selected in tree forests types of Dabie Mountain, East China. 44 soil samples from 12 plots (100 m^2^) at least 100 m apart in the two sites were collected from the organic horizon (O horizon) and the mineral horizon (M horizon) in the three forest types including *Cunninghamia*, *Pinus*, and broad-leaved forest based on five-point sampling method using soil borer (Ф 3 cm) and spade after removing litter of the upper layers^24^. Soil samples were filtered through a 2-mm sieve to remove roots and stones, and then divided into two parts. On part was air-dried to analyze soil organic matter. The other part was stored at − 80 °C for bacterial communities. Details of the environmental factors and soil chemical properties were described in our previous study^24^.

### Soil physical and chemical properties

pH was determined with a soil–water solution with a pH meter. Soil available phosphorus (AP) was analyzed using the Bray 1 method^45^. The content of soil total organic carbon (TOC) content and the total organic nitrogen (TON) were determined using the high-temperature catalytic combustion method^6^. The content of available potassium (AK) was determined with ammonium acetate and measured with a flame photometer. The content of ammonium nitrogen (AHN) was measured using the alkaline hydrolysis diffusion method.

### Illumina sequencing analysis of 16S rRNA genes

Total bacterial genomic DNA of 44 fresh soil samples were extracted from 0.3 g using the E.Z.N.A™ Mag-Bind Soil DNA Kit (M5635-02; OMEGA, USA). The quality and concentration of the extracted DNA were assessed using the Qubit 3.0 DNA Kit. The V3–V4 variable region of bacterial 16S rRNA genes was amplified by universal primers 341F and 805R and sequencing were performed in a volume of 30 µl^46^: at the first step, 3 min at 94 °C for initialization; 5 cycles of 30 s at 94 °C for denaturation, 20 s at 45 °C for annealing, and 30 s at 65 °C for extension; followed by 20 cycles of 20 s at 94 °C for denaturation, 20 s at 55 °C for annealing, and 30 s at 72 °C for extension; followed by 5 min at 72 °C for final elongation. At the second step, 3 min at 95 °C for initialization; 5 cycles of 20 s at 94 °C for denaturation, 20 s at 55 °C for annealing, and 30 s at 72 °C for extension, and 30 s at 72 °C for extension; followed by 5 min at 72 °C for final elongation. PCR products were then sequenced using the MiSeq v2 reagent cartridge on the Illumina MiSeq platform: 3 min at 95 °C for initialization; 5 cycles of 20 s at 94 °C for denaturation, 20 s at 55 °C for annealing, and 30 s at 72 °C for extension; followed by 5 min at 72 °C for final elongation. MagicPure Size Selection DNA Beads was used to purify and recover the PCR products. Qubit 3.0 DNA Kit was used to determine the quality of PCR products.

### Sequence analysis

Raw sequences ranging from 31,623 to 41,474 bp reads per sample were processed following the Quantitative Insights Into Microbial Ecology (QIIME) software package^47^. Raw sequences were first trimmed at a length of 200 bp using cutadapt (v1.10), Pear (v1.9.4), and Prinseq-lite (v0.20.4). Primer sequences were removed using cutadaptand then tail region sequences were removed using a slightly lower mass value with Prinseq-lite V0.20.4^48,49^. Paired-end reads were merged using Pear(v1.9.4). N-part sequences, short sequences, and low complexity sequences of each sequence were removed using the sliding window method. After removing the singleton and chimeric sequences, the high-quality sequences with ≥ 97% similarity were assigned operational taxonomic units (OTUs) using Usearch (v5.2.236)^50^. All samples were rarefied to minimum sequence read depth (30351) before downstream analysis. The raw sequence files and associated metadata were deposited in the Genome Sequence Archivein National Genomics Data Center (Database Resources of the National Genomics Data Center, 2022)^51^, China National Center for Bioinformation/Beijing Institute of Genomics, Chinese Academy of Sciences (GSA: CRA007977).

### Statistical analyses

All taxonomies of bacterial OTUs were assigned by RDP Classifier (v2.12) using a naïve Bayesian assignment with a mini-confidence of 0.8 which was considered to represent phylum, class, order, family, genus, and species levels. The bacterial community composition in the two soil horizons of three forest types were analyzed in VennDiagram package. The relationships among all samples were analyzed by Hierarchical clustering analysis with arithmetic average based on Bray–Curtis distance in vegan package. Bacterial alpha-diversity in the two soil horizons and three forest types, including richness, Chao1, Shannon, and Simpson index were assisted using Mothur (v1.30.1)^52^. Soil horizons and forest types effect on beta-diversity were analyzed using per-mutational multivariate analysis of variance based on weighted-UniFrac in the vegan package^53^. The significant differences of bacterial diversity and community composition in the two horizons and three forest types were determined using Stamp (v2.1.3, *P* ≤ 0.05). The significant differences among samples were assisted using Linear discriminant analysis effect size (LEfSe). In addition, the multivariate relationships between bacterial composition and soil horizon and forest type respectively were performed with Principal coordinate analysis (PCoA) and nonmetric multidimensional scaling (NMDS). The effect of soil organic matter on bacterial community in all samples was analyzed with multiple linear regression analysis and redundancy analysis (RDA). Soil horizons and forest types effect on beta-diversity were calculated using permutational multivariate analysis of variance and similarities function (ANOSIM) based on Bray–Curtis distance in the vegan package^54^. The soil bacterial functions were assessed with Kyoto Encyclopedia of Genes and Genomes (KEGG) using reconstruction of unobserved states (PICRUSt)^55–58^. All statistical analyses were run in *R* (4.2.0).

### Supplementary Information

Supplementary Legends.

Supplementary Information 1.

Supplementary Information 2.

Supplementary Information 3.

Supplementary Information 4.

## Data Availability

The data that support the findings of this study are openly available in China National Center for Bioinformation/Beijing Institute of Genomics, Chinese Academy of Sciences [GSA: CRA007977].

## References

[1] Tian J, He N, Hale L, Niu S, Zhou J (2018). Soil organic matter availability and climate drive latitudinal patterns in bacterial diversity from tropical to cold temperate forests. Funct. Ecol..

[2] Isobe K, Bouskill NJ, Brodie EL, Sudderth EA, Martiny JB (2020). Phylogenetic conservation of soil bacterial responses to simulated global changes. Philos. Trans. R. Soc. B.

[3] Ushio M, Wagai R, Balser TC, Kitayama K (2008). Variations in the soil microbial community composition of a tropical montane forest ecosystem: Does tree species matter?. Soil Biol. Biochem..

[4] Wang H (2019). Experimental warming reduced topsoil carbon content and increased soil bacterial diversity in a subtropical planted forest. Soil Biol. Biochem..

[5] Wall DH, Nielsen UN, Six J (2015). Soil biodiversity and human health. Nature.

[6] Zhao F (2019). Change in soil bacterial community during secondary succession depend on plant and soil characteristics. CATENA.

[7] Prober SM (2015). Plant diversity predicts beta but not alpha diversity of soil microbes across grasslands worldwide. Ecol. Lett..

[8] Nakayama M, Imamura S, Taniguchi T, Tateno R (2019). Does conversion from natural forest to plantation affect fungal and bacterial biodiversity, community structure, and co-occurrence networks in the organic horizon and mineral soil?. Forest Ecol. Manage..

[9] Liu J (2018). Effects of tree species and soil properties on the composition and diversity of the soil bacterial community following afforestation. Forest Ecol. Manage..

[10] Pei ZQ (2016). Soil and tree species traits both shape soil microbial communities during early growth of Chinese subtropical forests. Soil Biol. Biochem..

[11] Chodak M, Klimek B, Niklińska M (2016). Composition and activity of soil microbial communities in different types of temperate forests. Biol. Fertil. Soils.

[12] Lu JZ, Scheu S (2021). Response of soil microbial communities to mixed beech-conifer forests varies with site conditions. Soil Biol. Biochem..

[13] Shen C (2020). Contrasting patterns and drivers of soil bacterial and fungal diversity across a mountain gradient. Environ. Microbiol..

[14] Wan X (2015). Soil C:N ratio is the major determinant of soil microbial community structure in subtropical coniferous and broadleaf forest plantations. Plant Soil.

[15] Fierer N, Jackson RB (2006). The diversity and biogeography of soil bacterial communities. Proc. Natl. Acad. Sci..

[16] Liu W (2020). Critical transition of soil bacterial diversity and composition triggered by nitrogen enrichment. Ecology.

[17] Tan W, Wang J, Bai W, Qi J, Chen W (2020). Soil bacterial diversity correlates with precipitation and soil pH in long-term maize cropping systems. Sci. Rep.-Uk.

[18] Fierer N (2012). Comparative metagenomic, phylogenetic and physiological analyses of soil microbial communities across nitrogen gradients. Isme J..

[19] Wang C, Liu D, Bai E (2018). Decreasing soil microbial diversity is associated with decreasing microbial biomass under nitrogen addition. Soil Biol. Biochem..

[20] Liang X (2020). Lysogenic reproductive strategies of viral communities vary with soil depth and are correlated with bacterial diversity. Soil Biol. Biochem..

[21] Voříšková J, Brabcová V, Cajthaml T, Baldrian P (2014). Seasonal dynamics of fungal communities in a temperate oak forest soil. New Phytol..

[22] Nunan N, Schmidt H, Raynaud X (2020). The ecology of heterogeneity: Soil bacterial communities and C dynamics. Philos. Trans. R. Soc. B.

[23] Eilers KG, Debenport S, Anderson S, Fierer N (2012). Digging deeper to find unique microbial communities: The strong effect of depth on the structure of bacterial and archaeal communities in soil. Soil Biol. Biochem..

[24] Luo X (2021). Fungal community composition and diversity vary with soil horizons in a subtropical forest. Front. Microbiol..

[25] Hartemink A (2020). Soil horizon variation: A review. Adv. Agron..

[26] Plassart P (2019). Soil parameters, land use, and geographical distance drive soil bacterial communities along a European transect. Sci. Rep.-Uk.

[27] Li Y, Gong J, Liu J, Hou W, Moroenyane I (2022). Effects of different land use types and soil depth on soil nutrients and soil bacterial communities in a Karst Area, Southwest China. Soil Syst..

[28] van Leeuwen J (2017). Effects of land use on soil microbial biomass, activity and community structure at different soil depths in the Danube floodplain. Eur. J. Soil Biol..

[29] Wu B (2022). Effects of environmental factors on soil bacterial community structure and diversity in different contaminated districts of Southwest China mine tailings. Sci. Total Environ..

[30] Guo Q, Yan L, Korpelainen H, Niinemets Ü, Li C (2019). Plant-plant interactions and N fertilization shape soil bacterial and fungal communities. Soil Biol. Biochem..

[31] Ma S (2019). Plant species identity and soil characteristics determine rhizosphere soil bacteria community composition in European temperate forests. Fems Microbiol. Ecol..

[32] Wang J (2020). Plant functional traits regulate soil bacterial diversity across temperate deserts. Sci. Total Environ..

[33] Wardle DA (2004). Ecological linkages between aboveground and belowground biota. Science.

[34] Zhong Z (2020). Soil bacteria and fungi respond differently to plant diversity and plant family composition during the secondary succession of abandoned farmland on the Loess Plateau, China. Plant Soil.

[35] Urbanova M, Snajdr J, Baldrian P (2015). Composition of fungal and bacterial communities in forest litter and soil is largely determined by dominant trees. Soil Biol. Biochem..

[36] Ding GC (2013). Changes of soil bacterial diversity as a consequence of agricultural land use in a semi-arid ecosystem. PLoS One.

[37] Sansupa C (2021). Can we use functional annotation of prokaryotic taxa (FAPROTAX) to assign the ecological functions of soil bacteria?. Appl. Sci..

[38] Bell T (2005). The contribution of species richness and composition to bacterial services. Nature.

[39] Mukhopadhya I, Hansen R, El-Omar EM, Hold GL (2012). IBD—what role do Proteobacteria play?. Nat. Rev. Gastroenterol. Hepatol..

[40] Spain AM, Krumholz LR, Elshahed MS (2009). Abundance, composition, diversity and novelty of soil Proteobacteria. ISME J..

[41] Dedysh SN, Damsté JSS (2018). Acidobacteria. LS.

[42] Kielak AM (2016). The ecology of Acidobacteria: Moving beyond genes and genomes. Front. Microbiol..

[43] Erlacher A (2015). Rhizobiales as functional and endosymbiontic members in the lichen symbiosis of *Lobaria pulmonaria* L.. Front. Microbiol..

[44] Fei Y (2020). Response of soil enzyme activities and bacterial communities to the accumulation of microplastics in an acid cropped soil. Sci. Total Environ..

[45] Bray RH, Kurtz L (1945). Determination of total, organic, and available forms of phosphorus in soils. Soil Sci..

[46] Palansooriya KN (2022). Biochar alters chemical and microbial properties of microplastic-contaminated soil. Environ. Res..

[47] Caporaso JG, Kuczynski J, Stombaugh J, Bittinger K, Bushman FD (2010). QIIME allows analysis of high-throughput community sequencing data. Nat. Methods.

[48] Schmieder R, Edwards R (2011). Quality control and preprocessing of metagenomic datasets. Bioinformatics.

[49] Zhang J, Kobert K, Flouri T, Stamatakis A (2014). PEAR: A fast and accurate illumina paired-end reAd mergeR. Bioinformatics.

[50] Edgar RC (2010). Search and clustering orders of magnitude faster than BLAST. Bioinformatics.

[51] Chen T, Chen X, Zhang S, Zhu J, Tang B (2021). The genome sequence archive family: Toward explosive data growth and diverse data types. Genom. Proteom. Bioinform..

[52] Schloss PD (2009). Introducing mothur: Open-source, platform-independent, community-supported software for describing and comparing microbial communities. Appl. Environ. Microbiol..

[53] Lozupone C, Lladser ME, Knights D, Stombaugh J, Knight R (2011). UniFrac: An effective distance metric for microbial community comparison. ISME J..

[54] Yang T (2017). Fungal community assemblages in a high elevation desert environment: Absence of dispersal limitation and edaphic effects in surface soil. Soil Biol. Biochem..

[55] Langille MGI, Zaneveld J, Caporaso JG, McDonald D, Knights D (2013). Predictive functional profiling of microbial communities using 16S rRNA marker gene sequences. Nat. Biotechnol..

[56] Kanehisa M, Goto S (2000). KEGG: Kyoto Encyclopedia of Genes and Genomes. Nucleic Acids Res..

[57] Kanehisa M (2019). Toward understanding the origin and evolution of cellular organisms. Protein Sci..

[58] Kanehisa M, Furumichi M, Sato Y, Kawashima M, Ishiguro-Watanabe M (2023). KEGG for taxonomy-based analysis of pathways and genomes. Nucleic Acids Res..

