# Supplementary material for: Soil horizons regulate bacterial community structure and functions in Dabie Mountain of the East China
Source: Sci Rep. 2023 Sep 22;13:15866. doi: 10.1038/s41598-023-42981-7 (PMC10517015; doi:10.1038/s41598-023-42981-7)
Supplement: Supplementary file 1 — Supplementary Legends. [file 41598_2023_42981_MOESM1_ESM.docx]

**Figs.1 (a)** Bacterial abundance based on the hierarchy genus and phylum in in all samples at Dabie Mountain. **(b)** Bacterial community composition based on genus level in the two soil horizon.

**Figs. 2** Effects of soil horizon and forest types on fungal richness index (a) (c) and Shannon index (b) (d) at Tiantangzhai and Yaoluoping Nature Reserve. The letter means the significant difference in bacterial richness and diversity between two groups. TO represents the O horizon at Tiantangzhai Nature Reserve.

**Figs.3** Significantly altered bacterial communities among three forest types in the soil organic matter horizon as measured by the response ratio method at the 95% confidence interval (Welch’s t-test) at Yaoluoping (a) (b) (c) and Tiantangzhai Nature Reserve (d) (e) (f). Only the 25 with the lowest *P* value are displayed.

**Figs.4** Significantly altered bacterial communities among three forest types in the mineral matter mixed with some humus horizon as measured by the response ratio method at the 95% confidence interval (Welch’s t-test) at Yaoluoping (a) (b) (c) and Tiantangzhai Nature Reserve (d) (e) (f). Only the 25 with the lowest *P* value are displayed.
