# Supplementary material for: Soil horizons regulate bacterial community structure and functions in Dabie Mountain of the East China
Source: Sci Rep. 2023 Sep 22;13:15866. doi: 10.1038/s41598-023-42981-7 (PMC10517015; doi:10.1038/s41598-023-42981-7)
Supplement: Supplementary file 2 — Supplementary Information 1. [file 41598_2023_42981_MOESM2_ESM.docx]

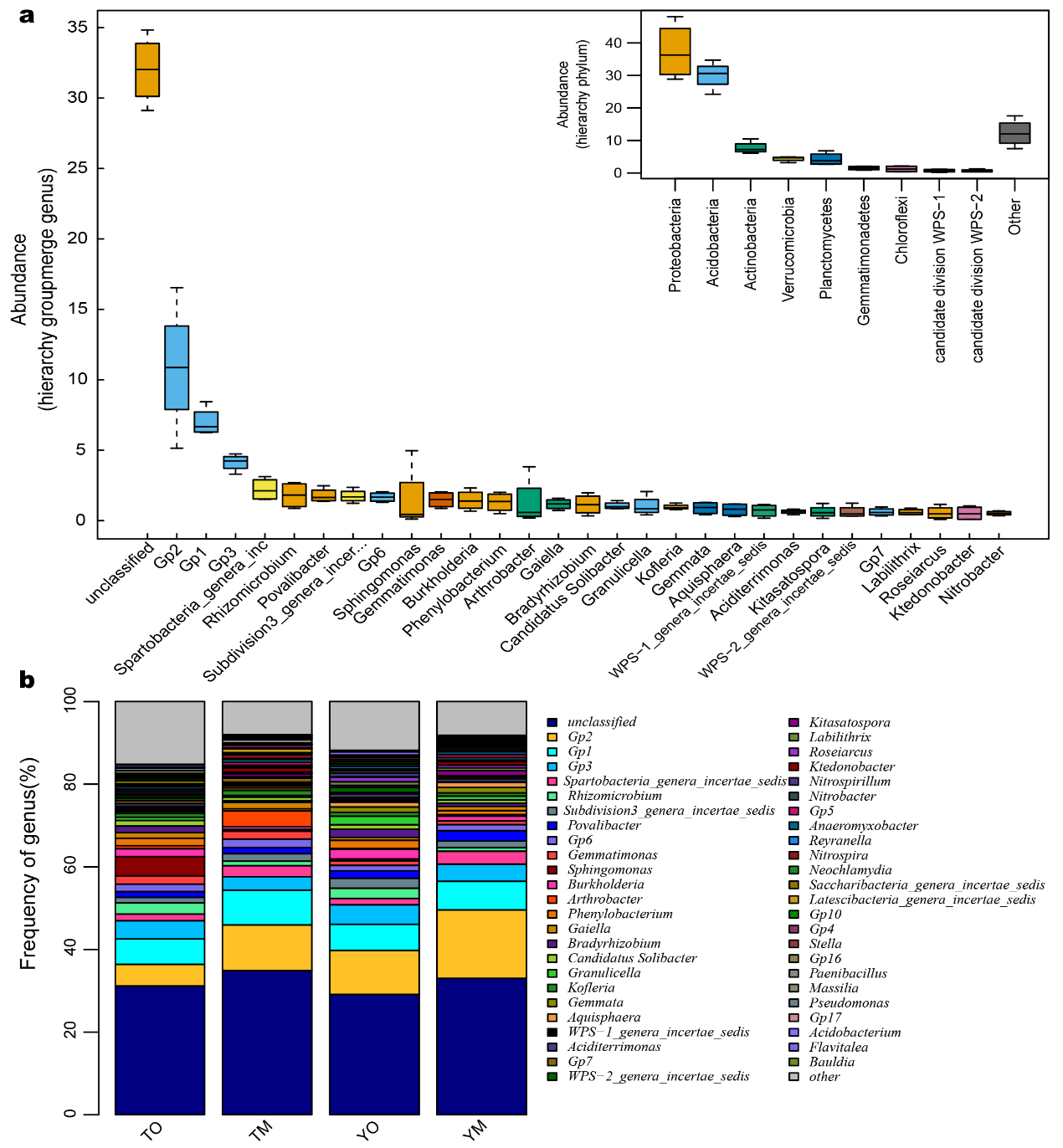


**Figs.1 (a)** Bacterial abundance based on the hierarchy genus and phylum in in all samples at Dabie Mountain. **(b)** Bacterial community composition based on genus level in the two soil horizon. *T*: Tiantangzhai Nature Reserve; *Y*: Yaoluoping Nature Reserve; *O*: the soil organic matter; *M*: the mineral matter mixed with some humus.
