# Supplementary material for: Soil horizons regulate bacterial community structure and functions in Dabie Mountain of the East China
Source: Sci Rep. 2023 Sep 22;13:15866. doi: 10.1038/s41598-023-42981-7 (PMC10517015; doi:10.1038/s41598-023-42981-7)
Supplement: Supplementary file 3 — Supplementary Information 2. [file 41598_2023_42981_MOESM3_ESM.docx]

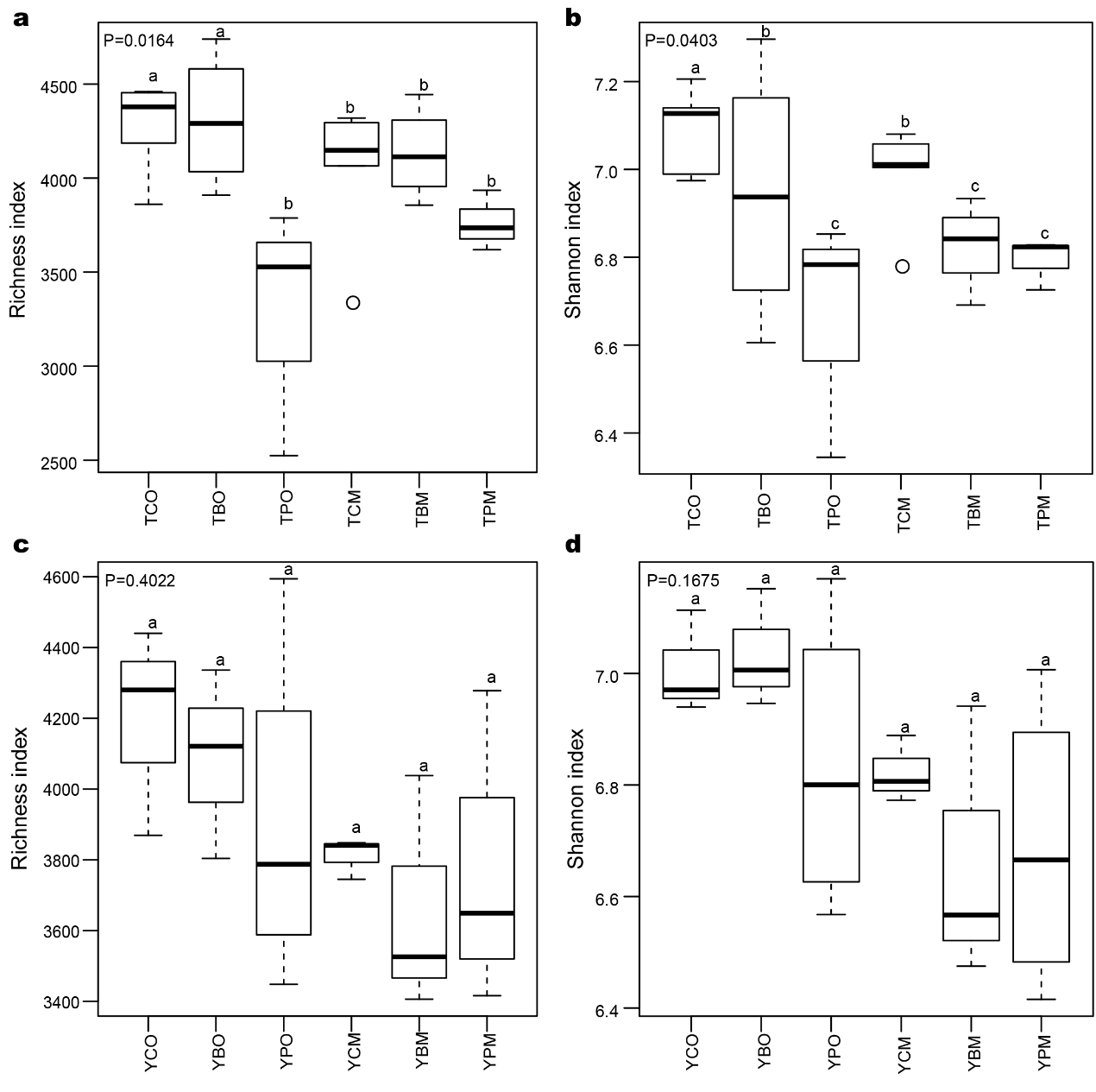


**Figs. 2** Effects of soil horizon and forest types on fungal richness index (a) (c) and Shannon index (b) (d) at Tiantangzhai and Yaoluoping Nature Reserve. The letter means the significant difference in bacterial richness and diversity between two groups. TO represents the O horizon at Tiantangzhai Nature Reserve. *T*: Tiantangzhai Nature Reserve; *Y*: Yaoluoping Nature Reserve; *O*: the soil organic matter; *M*: the mineral matter mixed with some humus; *C*: *Cunninghamia* forest; *B*: broad-leaved forest; *P*: *Pinus* forest.
