# Supplementary material for: Soil horizons regulate bacterial community structure and functions in Dabie Mountain of the East China
Source: Sci Rep. 2023 Sep 22;13:15866. doi: 10.1038/s41598-023-42981-7 (PMC10517015; doi:10.1038/s41598-023-42981-7)
Supplement: Supplementary file 5 — Supplementary Information 4. [file 41598_2023_42981_MOESM5_ESM.docx]

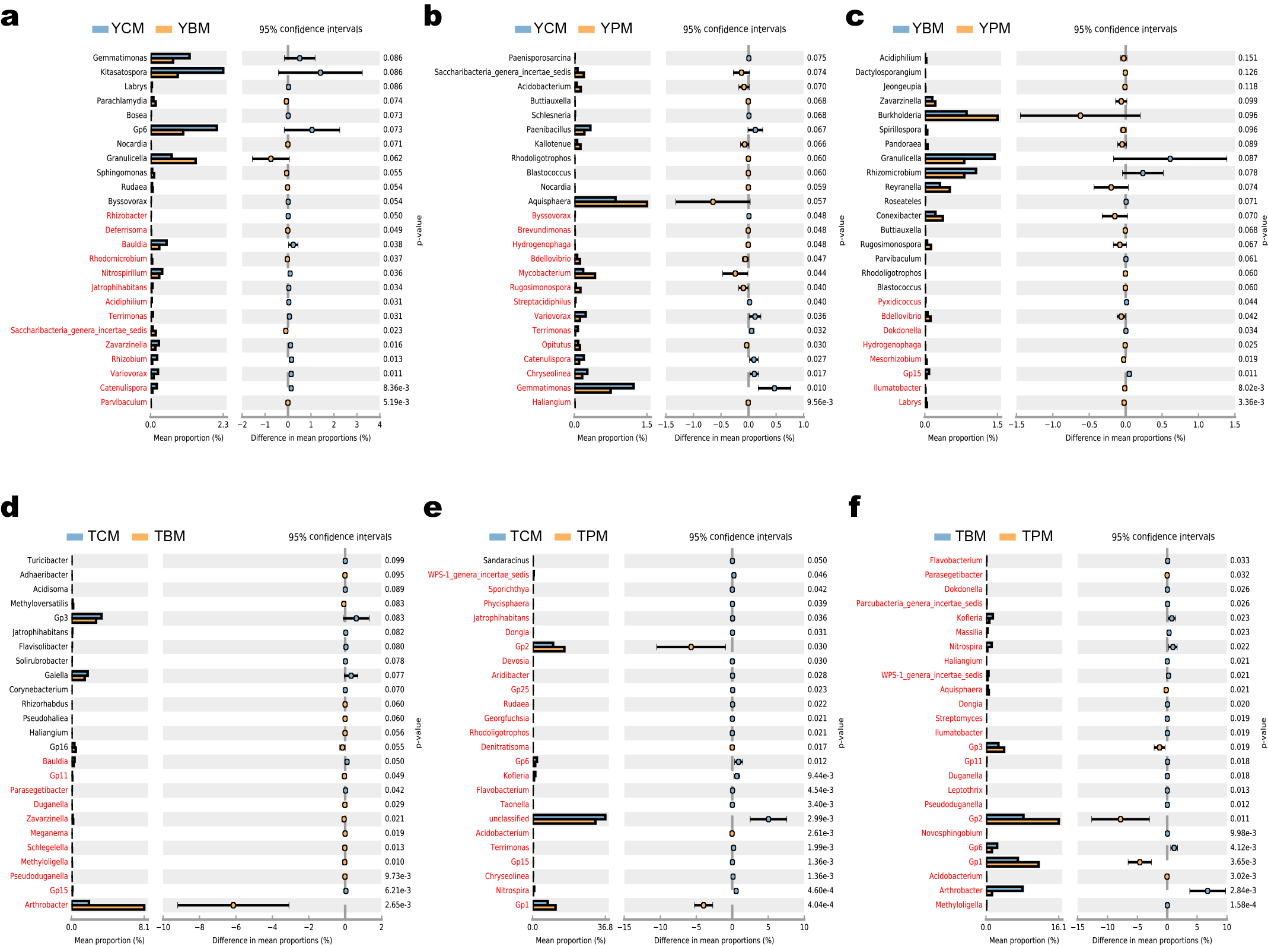


**Figs.4** Significantly altered bacterial communities among three forest types in the mineral matter mixed with some humus horizon as measured by the response ratio method at the 95% confidence interval (Welch’s t-test) at Yaoluoping (a) (b) (c) and Tiantangzhai Nature Reserve (d) (e) (f). Only the 25 with the lowest *P* value are displayed. *T*: Tiantangzhai Nature Reserve; *Y*: Yaoluoping Nature Reserve; *M*: the mineral matter mixed with some humus; *C*: *Cunninghamia* forest; *B*: broad-leaved forest; *P*: *Pinus* forest.
